# Synthesis and Characterization of Nanohydroxyapatite-Gelatin Composite with Streptomycin as Antituberculosis Injectable Bone Substitute

**DOI:** 10.1155/2019/7179243

**Published:** 2019-06-25

**Authors:** Dyah Hikmawati, Hendita N. Maulida, Alfian P. Putra, Aniek S. Budiatin, Ardiyansyah Syahrom

**Affiliations:** ^1^Department of Physics, Faculty of Science and Technology, Universitas Airlangga, Surabaya 60115, Indonesia; ^2^Biomedical Engineering, Department of Physics, Faculty of Science and Technology, Universitas Airlangga, Surabaya 60115, Indonesia; ^3^Department of Clinical Pharmacy, Faculty of Pharmacy, Universitas Airlangga, Surabaya 60286, Indonesia; ^4^Applied Mechanics and Design, School of Mechanical Engineering, Faculty of Engineering, Universiti Teknologi Malaysia (UTM), Johor Bahru, 81310, Malaysia; ^5^Medical Devices and Technology Centre (MEDITEC), Institute of Human Centred and Engineering (iHumEn), Universiti Teknologi Malaysia (UTM), Johor Bahru, 81310, Malaysia

## Abstract

The most effective treatment for spinal tuberculosis was by eliminating the tuberculosis bacteria and replacing the infected bone with the bone graft to induce the healing process. This study aims to synthesize and characterize nanohydroxyapatite-gelatin-based injectable bone substitute (IBS) with addition of streptomycin. The IBS was synthesized by mixing nanohydroxyapatite and 20 w/v% gelatin with ratio of 40:60, 45:55, 50:50, 55:45, 60:40, 65:35, 70:30, and 75:25 ratio and streptomycin addition as antibiotic agent. The mixture was added by hydroxypropyl methylcellulose as suspending agent. FTIR test showed that there was a chemical reaction occurring in the mixture, between the gelatin and streptomycin. The result of injectability test showed that the highest injectability of the IBS sample was 98.64% with the setting time between 30 minutes and four hours after injection on the HA scaffold that represents the bone cavity and coat the pore scaffold. The cytotoxicity test result showed that the IBS samples were nontoxic towards BHK-21 fibroblast cells and human hepatocyte cells since the viability cell was more than 50% with significant difference (*p*-value<0.05). The acidity of the IBS was stable and it was sensitive towards* Staphylococcus aureus* with significantly difference (*p*-value<0.05). The streptomycin release test showed that the streptomycin could be released from the IBS-injected bone scaffold with release of 2.5% after 4 hours. All the results mentioned showed that IBS was suitable as a candidate to be used in spinal tuberculosis case.

## 1. Introduction

Tuberculosis is one of infectious diseases which has high mortality level in the world. More than one million people in the world die because of this disease [1]. In 2016, World Health Organization reported that this disease reached a number of 10.4 million cases and 34% of them occurred in Southeast Asia. One of them was Indonesia [1, 2]. 5-10% of them occurred on the bone and joint, especially in the spine [3]. The treatment of this case is usually by using antituberculosis drugs through intravenous and intramuscular [4]. The most effective treatment could be performed by eliminating the tuberculosis bacteria directly and replacing the infected bone with the bone graft or filler to induce the healing process and spinal stability [3, 4]. Spinal surgery is needed to implement this method. Instead of only using bone graft to fill the defect, a drug is also embedded or inserted to improve its function [5, 6].

Hydroxyapatite is one of the materials that could be used as bone graft [7]. This material has biocompatible and bioactive properties which is beneficial for restoring the bone defect due to the bone tuberculosis. One drawback of this material is brittle. Thus, it was widely combined with the other materials to form a composite, especially polymer, to obtain the desired properties. One of them is gelatin. This material is already globally used in medical field, especially for drug capsule. This material is also biocompatible, biodegradable, and nontoxic. This material is extensively used as a filler due to its ease to set [7, 8].

There are several cases of bone defect that requires the bone filler to fill irregular defect and one of the solutions is using the bone filler that is in a form of paste or suspension. This is commonly called injectable bone substitute (IBS). This method could reach the sharp defect on the one and set in the site of defect. By using a suspending agent like hydroxyl propyl methyl cellulose (HPMC), the IBS could be synthesized as mentioned by Weiss et al. (2007) and Shen et al. (2014) in their study [9–11]. The addition of antibiotic such streptomycin could help the healing process in the spinal tuberculosis case [12].

This study was focused on the synthesis and characterization of the IBS based on hydroxyapatite-gelatin with the addition of streptomycin for spinal tuberculosis case. The characterization performed here was FTIR test to observe the functional group of the sample, the injectability test to know the ability of the sample extruded from the syringe, and the setting time test on hydroxyapatite scaffold to observe the ability of the sample to set on the proper substrate. The biological characterization in this study consisted of cytotoxicity test by using MTT assay, the acidity test to know its stability in the SBF solution, and the antibacterial test by using* Staphylococcus aureus*. The streptomycin release test was used to obtain the release profile of streptomycin by first determining the standard curve of streptomycin to show the correlation between the streptomycin concentration and absorption.

## 2. Materials and Methods

Nanohydroxyapatite used in this study was obtained from Badan Tenaga Nuklir Nasional (BATAN) Jakarta, Indonesia, originated from the fish scales. The gelatin was derived from cow skin purchased from 150 bloom Rousselot (Guangdong, China). The streptomycin sulphate (powder for injection) was obtained from PT. Meiji Indonesia. The hydroxypropyl methylcellulose (HPMC) was obtained from Sigma Aldrich H7509.

The materials used for characterization were hydroxyapatite scaffold from Tissue Bank General Hospital Dr. Soetomo (Surabaya, Indonesia) for setting time test, NaCl, NaHCO_3_, KCl, K_2_HPO_4_.3H_2_O, MgCl_2_.6H_2_O, HCl, CaCl_2_.2H_2_O, Na_2_SO_4_ dan (HOCH_2_)_3_CNH_2_ for SBF solution and* Staphylococcus aureus* for antibacterial test.

The tools used were freezer and lyophilizer, 10 cc syringe, viscotester VT-04F RION, pH meter Benchtop OAKTON, Scanning Electron Microscope (SEM) FEI Inspect S50 Japan, and UV-Vis Spectrometer.

### 2.1. Synthesis of HA-GEL-Streptomycin Injectable Bone Substitute (IBS)

The injectable bone substitute (IBS) was synthesized by dissolving 20 w/v% gelatin (GEL) in deionized water at temperature of 40°C for one hour. The hydroxyapatite (HA) powder was added to that solution with several ration of HA and GEL (of 40:60, 45:55, 50:50, 55:45, 60:40, 65:35,70:30 and 75:25). 10wt% streptomycin was added in the mixture. Meanwhile, HPMC 4% w/v was dissolved in distilled water at 90°C and then added to solution of gelatin, hydroxyapatite, and streptomycin at 40°C and stirred for six hours to produce a white IBS.

### 2.2. Sample Characterization

The characterization for IBS in this study consisted of Fourier Transform Infrared (FTIR) Test, Injectability test, setting time test, cytotoxicity test, acidity test, and antibacterial test. The FTIR test was aimed at observing that there was no interaction among the materials used in this study based on the functional groups. The sample with KBr was made into a pellet. The pellet was tested with a wavenumber range of 4000-400 cm^−1^.

The injectability test was aimed at observing the ability of the IBS to be extruded from a syringe within a range of time. The test used the method of Shen et al. (2014) by utilizing 10 cc syringe and a needle with an inner diameter of 1.2 mm [11]. The mass of IBS before and after the injection was measured. By using (1), the injectability of the IBS could be obtained. The test was repeated five times.

(1) Injectability%=mass  extruded  from  the  syringetotal  mass  before  injectionx100%

The setting time test was performed by using hydroxyapatite scaffold that has been freeze-dried as a substrate. The scaffold mass was measured to observe the changes that occurred after the setting of the IBS. The test was performed by injecting the IBS samples into the scaffold vertically. The time when penetration of the IBS started until the surface was fully dried was counted.

The four best samples based on two characterizations mentioned above were then continued to the biological characterization. The cytotoxicity test was performed by using MTT assay method which used 3-(4,5-dimethyl-2-thiazolyl)-2.5-diphenyl-2H-tetrazolium bromide (MTT). This substance would give the information of viability cell while it changed to formazan salt due to the activity of mitochondria of living cell. The cell used in this test was fibroblast cell from Baby Hamster Kidney (BHK-21). The optical density of formazan salt would be measured by using Elisa reader. The cell viability would be calculated by using (2). The materials were considered as not-toxic if the cell viability is more than 50% [13–15].

(2) Cell  Viability%=OD  treatment+OD  mediaOD  Cell+ODMediax100%

The best concentration of this test was then tested again by using human hepatocyte cells to evaluate its cytotoxicity further. Human hepatocyte cells are sensitive to toxic material so that it is used in cytotoxicity test. The human hepatocyte cells were cultured in DMEM media and incubated at 37°C. The cells were then observed under a microscope to make sure the cells were grown well. The medium was rinsed and changed with EDTA trypsin to detach the cells from the wall. The cells were incubated for 4 min and centrifuged for 4 min with 120 rpm to separate the cells and trypsin. DMEM solution was added to the cells and that mixture was inserted into each well. The cells in 96-microwell plate were incubated for 24 at temperature of 37°C. The DMEM solution was changed with a new 200 *μ*l DMSO carefully. Then, the tested sample was inserted to each well with 3 replications. The sample was then incubated for 48 h at temperature of 37°C. After 48 h, the sample in each well was taken and MTT solution was added to each well. The well plate was incubated 4 h. Then, the medium was taken out and 200 *μ*l DMSO was added to each well. The cells were read by using Elisa reader at wavenumber of 560 nm and 750 nm [16]. The cell viability was then determined by using (3).

(3) $$\begin{array}{c} Cell\;\;Viability\left(\;\%\;\right)=\frac{Sample\;\;Absorbance}{Control\;\;Absorbance}x100\% \end{array}$$

The acidity test was used to observe the stability of the IBS acidity when it met the body fluid. In this test, the Simulated Body Fluid (SBF) was used. The SBF contains NaCl, NaHCO_3_, KCl, K_2_HPO_4_.3H_2_O, MgCl_2_.6H_2_O, HCl, CaCl_2_.2H_2_O, Na_2_SO_4_ dan (HOCH_2_)_3_CNH_2_. The acidity of the IBS was measured every 12 hour for 7 days.

The antibacterial test was used to observe the ability of the IBS to inhibit the bacteria from growing by observing the bacterial inhibition zone diameter.* Staphylococcus aureus* (SA) was suspended in 9 mL Tryptic Soy Broth (TSB) and cultured in the incubator at temperature of 60°C for 24 hours. The nutrient agar was prepared as a medium for bacteria. IBS paste was put in wells agar medium with four repetitions and inhibition zone diameter was observed after incubation for 24 hours.

The streptomycin release test was conducted to observe the amount of the streptomycin that was released from the IBS over time. The best HA:GEL ratio was used in this test to observe the amount of streptomycin released from the IBS. PBS solution was used as a media of release. The IBS solution was injected in a bone scaffold to mimic the condition inside the bone. The IBS-injected scaffold was immersed in PBS solution. The 1 ml PBS solution was then taken and evaluated in UV-Vis Spectrophotometer to evaluate the streptomycin concentration. Before that, a standard curve was made by testing streptomycin at several different concentrations. Here, 4 concentrations (2%, 4%, 6%, and 8%) were used to determine the relation between concentration and absorption of the streptomycin.

The data was analyzed by using statistical test which was one-way ANOVA test with *α* of 95% and presented as its average and standard deviation.

## 3. Results and Discussion

### 3.1. Functional Group Test

**Figure 1:**
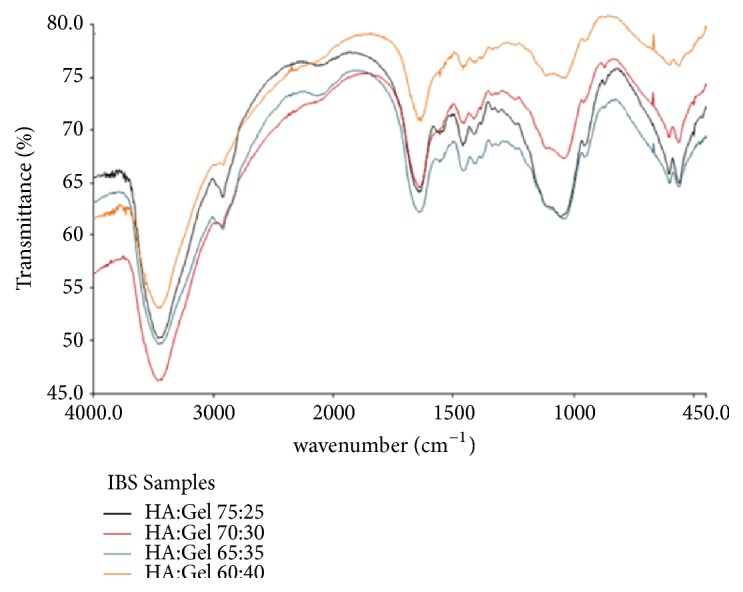
The FTIR result of IBS sample with several ratios of HA:GEL.

The IBS was synthesized in 8 variations of hydroxyapatite-gelatin ratio which were 40:60, 45:55, 50:50, 55:45, 60:40, 65:35, 70:30, and 75:25 ratios. The FTIR test was performed at several variations shown in Figure 1.

The result showed that there were several absorbance peaks related to some specific functional groups. The peak at wavenumber of 3467.5 cm^−1^ resembled the stretching vibration of hydroxyl group from the material used in this study, HA, GEL, HPMC, and streptomycin. The absorbance at wavenumber of 2929.47 cm^−1^ was the stretching vibration of C-CH_3_ which was the specific functional group from HPMC [7]. The peak at 1648.49 cm^−1^ showed the bonding between the carbonyl functional group originated from gelatin and the amine group from the streptomycin. Furthermore, the stretching vibration of P-O-C and phosphate functional group which was the specific functional groups from HA showed at wavenumber of 1041.48 cm^−1^ and 601.52 cm^−1^ [17]. There was no specific change in the functional groups happening in the sample which showed that there was no interaction between the materials used in this study. There was only physical interaction among the materials which were HA, GEL, HPMC, and streptomycin.

### 3.2. Injectability Test

**Figure 2:**
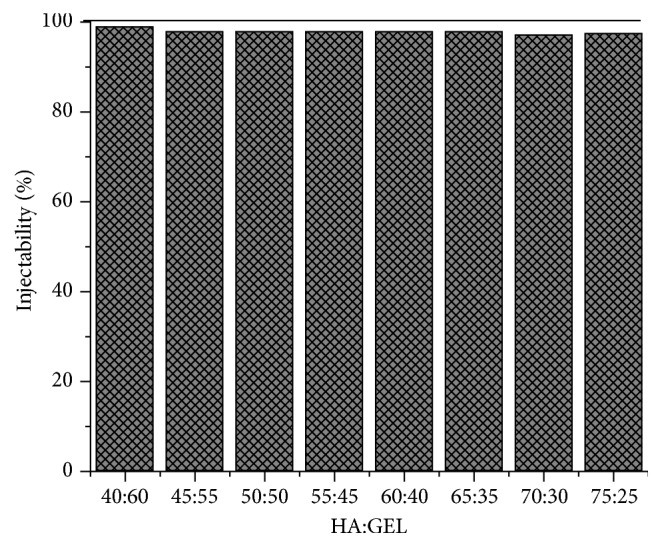
Injectability testing results of IBS.

The injectability test was performed by using a 10 cc syringe. The result of this test was shown in Figure 2. The eight samples of IBS had a very good ability in terms of injectability results which was approaching 100%. The best injectability was obtained by with HA-gelatin ratio at 40:60 (w/w) that is equal to 98.64%. This result was suited with the study of Shen et al. (2014) which synthesized IBS based on calcium phosphate and alendronate. Their result was 96.88 % of injectability with 3% alendronate [11]. Injectability was one of the most important properties in terms of IBS application since it should have high injectability to insert the IBS to the bone. Injectability is affected by the solvent and solute of the sample, the viscosity of the sample, and the syringe diameter. Both of the IBS and the syringe play important role in the IBS application [18].

### 3.3. Setting Time Test

**Figure 3:**
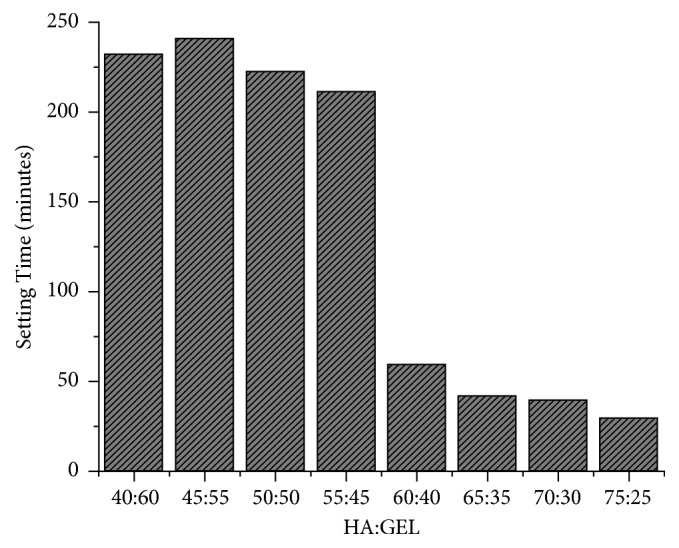
The setting time test results of IBS.

The setting time test was performed by using a freeze-dried HA scaffold as a model of human bone. The substrates had the same main component of the sample as the natural bone, such as the composition (hydroxyapatite) and the structure. The result of this test was shown in Figure 3. From the result of this result, there was a big difference between the sample with lower and higher hydroxyapatite content. The sample with 55% HA and below had longer setting time, while the sample with 60% HA and higher had shorter setting time. The bigger the composition of the hydroxyapatite in the mixture, the faster the setting time. Based on the study of Thai et al. (2010), they mentioned that the setting time of the IBS sample with calcium phosphate, calcium sulphate, HPMC, and citric acid was 30 minutes with 20% of citric acid and less than 10 minutes with 40% of citric acid [19]. The setting time of IBS less than 10 minutes could be applied in the defect of the small bone, such as carpal bone, while the other one could be used in the bigger bone, such as clavicle bone and its surrounding.

**Figure 4:**
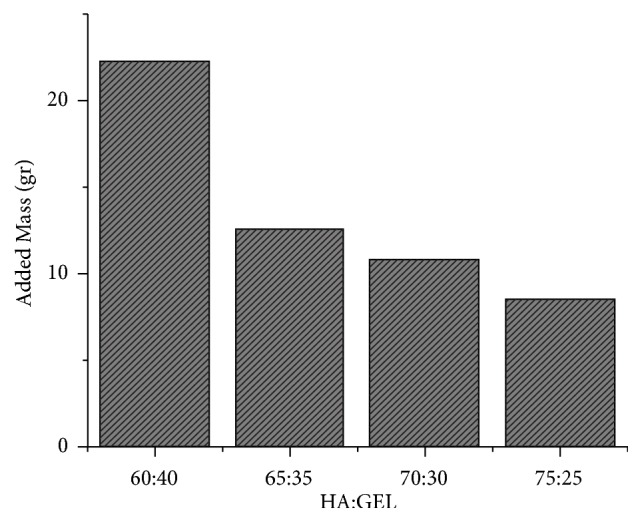
The added mass of the HA scaffold due to the presence of the IBS.

After the result in Figure 3, the faster variations was tested with the change in the mass due to the presence of the IBS in the HA scaffold. The result was shown in Figure 4. This change was caused by the scaffold HA that was synthesized by the freeze-dried method and it produced pores that allowed the IBS to infiltrate through the pores.

**Figure 5:**
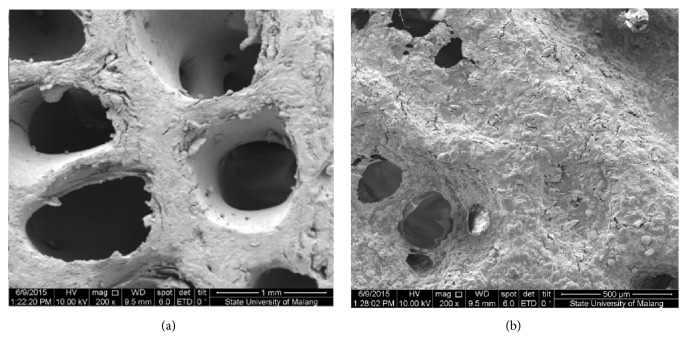
SEM of scaffold HA results (a) before the injection with IBS, magnification at 200x; (b) after injection of IBS and setting, magnification at 200x.

The changes in mass of the scaffold before and after the presence of the IBS were analyzed using Scanning Electron Microscope (SEM) as shown in Figure 5. From the SEM results, it depicted that HA scaffold surface was covered evenly by IBS. The pore size before injection showed the distribution of values in the range of 780.8-835.4 *μ*m and after scaffold injected by IBS in the range of 225.2 *μ*m. It might be concluded that the IBS could spread evenly into the pores of the scaffold and bound hydroxyapatite [2, 11]. Thus, IBS could be applied as bone substitute to fill the infected bone segments and vulnerable to further trigger the growth of new bone cells.

### 3.4. Acidity Test

**Figure 6:**
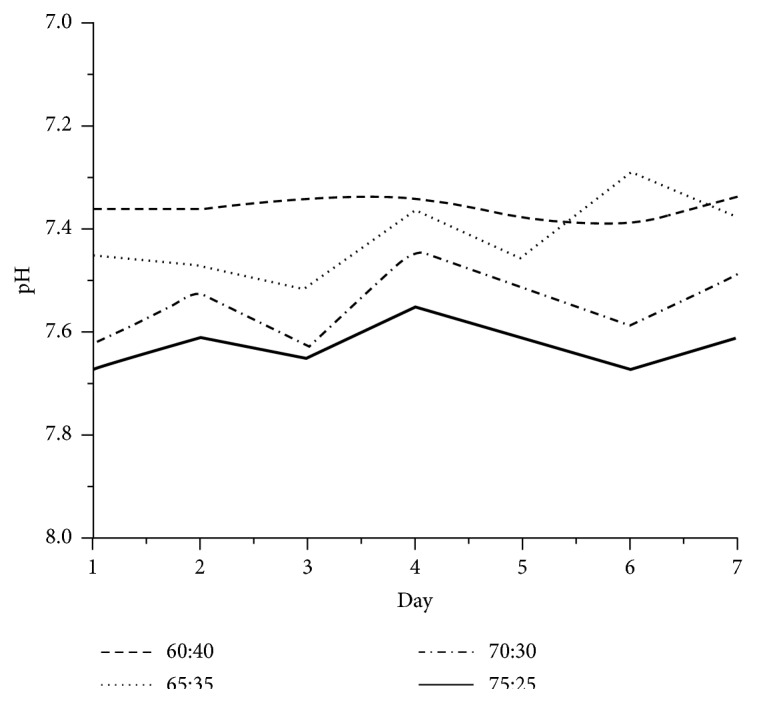
The acidity stability of the IBS sample with several HA:GEL ratios.

The acidity of the sample is one of the important points in the evaluation of the performance of IBS. The result in Figure 6 showed that the average pH of sample with HA-GEL ratio of 75:25, 70:30, 65:35, and 60:40 (w/w) were 7.62, 7.54, 7.41, and 7,35, respectively. The IBS needed pH more than 6 to be set in the bone. This result was still tolerable which could still be tolerated by the body and desired to give no pain effect [7, 20]. The acidity could also determine the setting process of the IBS since the IBS should be set at human body pH. Normally, the IBS should be set at pH of around 7.4. The sample with HA:GEL ratio of 65:35 was the most suitable one which had pH of 7.4 with little oscillation overtime.

### 3.5. Cytotoxicity Test

**Figure 7:**
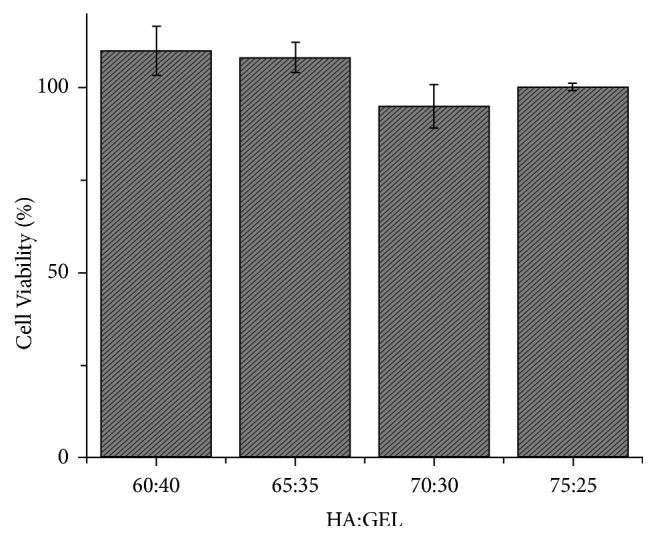
The cytotoxicity test result of the IBS sample with several HA:GEL ratios.

The cytotoxicity test was conducted by using fibroblast cells from Baby Hamster Kidney (BHK-21). The MTT enzyme would change to formazan salt with violet colour if it reacts to the mitochondria of the living cells. By measuring the intensity of this violet colour, we could obtain the cell viability. From this test, the result depicted that the IBS samples were nontoxic, because the cell viability was more than 50% shown in Figure 7. Besides that, this result also showed the percentage which was more than 100% [7, 13–15]. That result meant that the sample could promote proliferation of the cell. It indicated that the IBS could be the place for the osteoblast cells to grow [7, 14]. This result was significantly different with p-value=0.01857 (p-value<0.05).

The other cytotoxicity test was also performed to emphasize the effect of the sample towards the human cells. Human hepatocyte cells were used in this test. The best concentration of the sample based on the result of the other characterization was used in this which was the sample with HA:GEL ratio of 65:35. The result of this test showed that the cell viability of that sample was 93.69% with the amount of streptomycin of 100 mg/ml which equals 10%. This result showed that the sample was nontoxic, even after it was tested by using human hepatocyte cells [16]. Human hepatocyte cells in this test were successful to emphasize the cytotoxicity test that was using BHK-21 fibroblast cells. The further study of this sample by using human osteoblast cells is needed to observe the behaviour of the osteoblast cells when the IBS is applied.

### 3.6. Antibacterial Test

**Figure 8:**
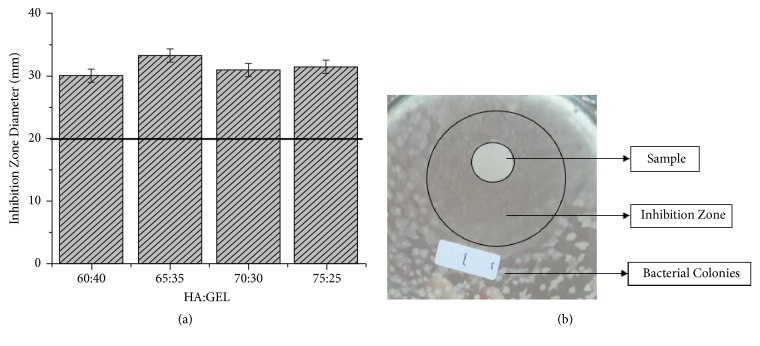
(a) The inhibition zone diameter of IBS in several HA:GEL ratios. (b) The inhibition zone around the sample that had streptomycin.

The antibacterial test result is depicted in Figure 8. The tested sample was paper with the released part of the IBS that was applied in the bone scaffold. In Figure 8(b), the inhibition zone was surrounding the sample and the white dots were the bacterial colonies. There was an inhibition zone around the sample with diameter of 28-33 mm from the initial well diameter which was 9 mm. Based on Balouiri et al. (2016), the level of bacterial resistance from a material could be evaluated by comparing the clear zone or inhibition zone diameter with the initial sample well diameter [21]. Based on that result, all the sample showed that they were sensitive to* Staphylococcus aureus* bacteria with inhibition zone more than 20 mm with significant difference,* p*-value = 0.00423 (*p*-value <0.05). The presence streptomycin in the mixture was the main reason of this antibacterial activity. The spinal tuberculosis is caused by* Mycobacterium tuberculosis*. These bacteria are neither gram-positive nor gram-negative bacteria since they have the characteristics of both types. The streptomycin could kill these bacteria by inhibition of protein synthesis of mycobacteria in the ribosome [22]. This process also happens in* S. aureus*.

### 3.7. Streptomycin Release Test

**Figure 9:**
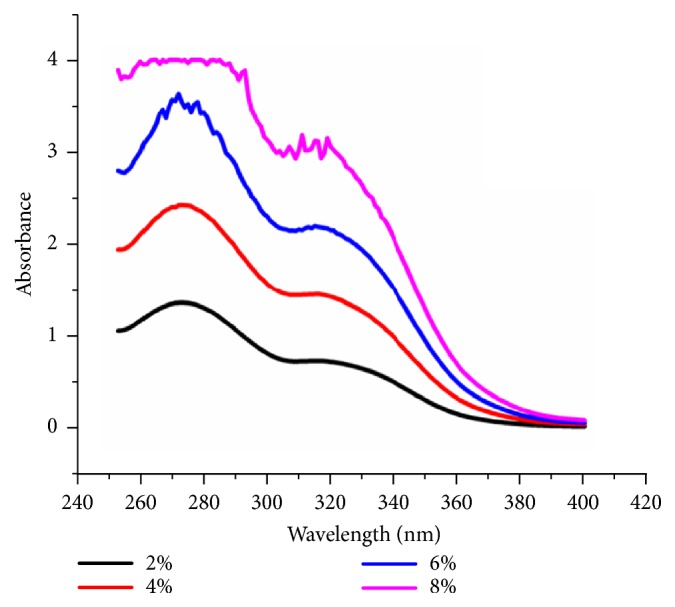
The streptomycin absorbance at wavelength of 250-400 nm.

The streptomycin release test was performed to observe the amount of streptomycin released from the IBS that was applied in the bone scaffold overtime. Streptomycin at several concentrations was first tested by using UV-Vis Spectrophotometer to determine the correlation between the streptomycin and the absorption. The result was shown in Figure 9.

**Figure 10:**
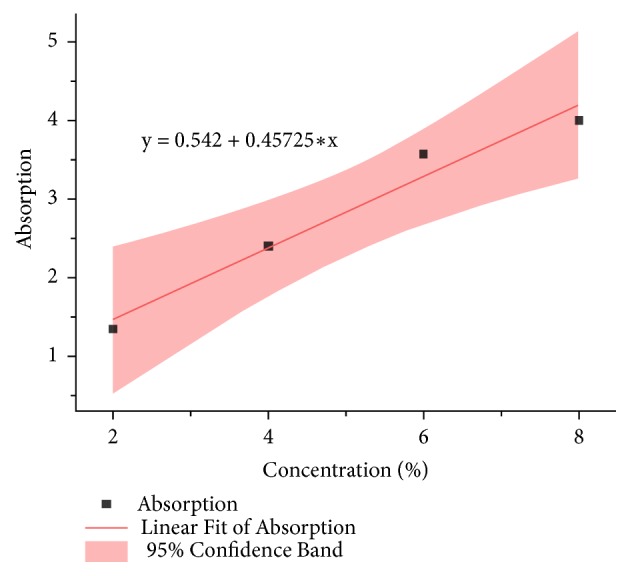
Standard curve of streptomycin with several concentrations.

The absorbance of streptomycin was specific at wavelength of 270 nm which was indicated by the peaks in each concentration. The absorbance at those peaks was taken to determine the standard curve and the correlation between the streptomycin concentration and its absorbance was obtained. The correlation of both parameters was then depicted in a linear curve with its equation in (4) with y being absorbance and x being concentration [23]. The streptomycin standard curve was presented at Figure 10.

(4) $$\begin{array}{c} y=0.457x+0.542 \end{array}$$

Thus, the concentration equation was obtained and showed in (5)

(5) $$\begin{array}{c} concentration=\frac{\left(absorbance-0.542\right)}{0.457} \end{array}$$

**Figure 11:**
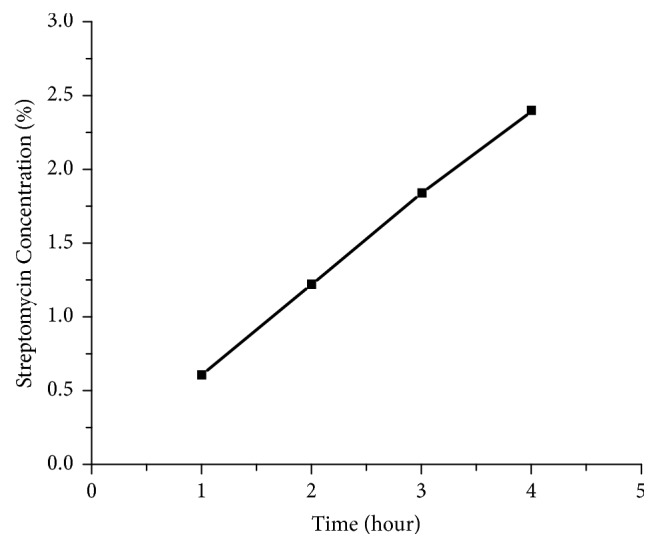
The streptomycin release profile overtime from the IBS-injected bone scaffold.

The standard curve was then used to determine the streptomycin concentration released by the IBS-injected bone scaffold. The result was shown in Figure 11. The release profile of streptomycin was quite linear and in 4 hour, the amount of streptomycin released was almost 2.5% [23]. The initial concentration was 10%. The result of cytotoxicity test showed that with the concentration of 10% it could still give high cell viability [14]. In the spinal tuberculosis case, the* Mycobacterium tuberculosis* could be killed by streptomycin. By the result of this test, the amount of streptomycin could release at desired concentration to its surrounding.

## 4. Conclusions

The IBS based on hydroxyapatite-gelatin-streptomycin has been synthesized. The result of FTIR test showed that there was a bonding between the streptomycin and the gelatin. The injectability test showed that all the samples had high injectability with the highest injectability of 98.64% and from setting time test, the sample with higher hydroxyapatite content had a faster setting time (one hour and less) when they were injected to the hydroxyapatite scaffold. The cytotoxicity test showed that the sample was nontoxic with the cell viability more than 50% from BHK-21 fibroblast cells and human hepatocyte cells. The acidity of all the samples was stable among 7.5. The antibacterial test showed that all the samples were sensitive to* Staphylococcus aureus* with inhibition zone more than 20 mm. The streptomycin was released at 2.5% after 4 hours.

## Data Availability

The data used to support the findings of this study are included within the article.
